# Ulipristal Acetate in Myomectomy Optimization in an Infertile Patient with Giant Myomas

**DOI:** 10.1155/2016/5135780

**Published:** 2016-08-10

**Authors:** Elena de la Fuente, María Dolores Borrás, Miriam Rubio, Nuria Abril

**Affiliations:** Department of Obstetrics and Gynecology, Hospital de Sagunto, Valencia, Spain

## Abstract

The use of ulipristal acetate (UPA) has been recently introduced in the treatment of uterine leiomyomas. This drug has proven useful to control menometrorrhagia and to reduce myoma size. In the case presented here, we show the benefits of UPA treatment in facilitating surgical removal of giant myomas in an infertile patient. In addition to myoma reduction and a better control of preoperative bleeding, the treatment with UPA reduced the duration and complexity of the surgery, as well as the area of uterine wall involved and the resulting scar. No side effects were observed and the patient became pregnant 6 months after the surgery and had a normal pregnancy and delivery. This case report shows the beneficial effects of UPA in the preoperative treatment of myomas which affect uterus function.

## 1. Introduction and Objective

Uterine leiomyomas affect 70–80% of females of childbearing age. Although most cases are asymptomatic, patients can present menometrorrhagia, pelvic pain, and infertility when the myomas are large or numerous or if their location is problematic (i.e., affecting the endometrial cavity). The reproductive prospects of the patient can be impaired when myomas are large or abundant because they can severely disrupt the structure of the uterus and its cavity [1, 2].

Besides surgical and nonsurgical techniques, the only medical treatments available until recently involved the use of GnRH agonists, and the levonorgestrel intrauterine device has also been applied as an out-of-label alternative [3].

The introduction of ulipristal acetate (UPA), a selective progesterone receptor modulator (SPRM), has opened new avenues for the control of uterine myomatosis. The administration of UPA has demonstrated efficacy in the control of menometrorrhagia and in the reduction of the size of myomas [3–5]. The reduction of myoma size, as well as, possibly, its tropism, is expected to improve the results of the myomectomy and the reproductive prospects of the patient.

The clinical case presented here is an example of how the use of this new therapeutic tool may improve the treatment of uterine myomatosis in patients wishing to become pregnant.

## 2. Clinical Case

A patient aged 34 (weight: 60 kg; height: 162 cm) with a gynecologic history of menometrorrhagia was examined after an 8-week interrupted gestation. The patient had become pregnant after 3 years of intending to become so. The echographic examination showed a gestational sac fully displaced by two intramural uterine myomas, 20 cm each, located in the uterine fundus and in the left anterior wall close to the uterine blood vessels of the same side and in the vicinity of the cervix. Total uterus volume was 28 × 27 × 22 cm.

After curettage, RMN was requested to confirm size and localization of the myomas. Since the patient wished to become pregnant, a myomectomy was considered. UPA (5 mg per day) was administered over a period of 8 weeks as a preoperative treatment, after which we observed by echographic examination that the diameters of both myomas had decreased in size from 20 to 18.4 cm (that at the uterine fundus) and to 17.8 cm (that at the left anterior wall). The echographic examination was suggestive of hyaline degeneration of the myomas. The patient reported amenorrhea and we observed an increase in hemoglobin levels of 1.2 g/dL and of hematocrit of 1.9% in this period.

A myomectomy by Pfannenstiel incision, eventless, with little blood loss, was performed on the patient. Myoma enucleation was extremely easy as we found an optimal angle of cleavage for decapsulation. We accomplished full removal of both myomas without the need to access the endometrial cavity.

Postsurgery recovery was satisfactory, and we observed a reduction in the hemoglobin level of 1.7 g/dL and hematocrit of 3.9%. After introduction of oral ferrotherapy (80 mg per day) for 2 months, the patient registered an increase of the hemoglobin level and hematocrit of 2.9 g/dL and 10%, respectively. The anatomopathological analysis confirmed that the myomas were 13 and 11 cm in diameter and weighted 567 and 430 g, respectively.

One month after the surgery, the uterus size was 7.7 × 7.6 × 4.7 cm as measured by vaginal echography. We noted a 2.5 cm postsurgical scar in the anterior side of the uterus. We advised the patient to avoid pregnancy for the following 9 months. Six months after initiating efforts to become pregnant, the patient achieved it spontaneously. Pregnancy was well controlled and without complications. Elective caesarean section in week 39 terminated the gestation, giving birth to a baby-girl weighting 3,390 g and Apgar score 10/10.

## 3. Discussion

The main objective of the pharmacological treatment recommended before myomectomy is to recover uterus function, especially in patients wishing to become pregnant. This can be achieved by controlling bleeding and reducing the size of myomas, which in turn improve the symptomatology and the quality of life of patients.

In the clinical case described here, the reduction of the size of the myoma and the control of bleeding before surgery were essential considering the impact of the size of the myomas in the endometrial cavity. The duration and complexity of the surgery, as well as the resulting scar, were probably reduced by the treatment with UPA, highlighting the important benefits provided by this drug. The patient did not report any side effects and became pregnant soon and without complications after the recommended resting period. Treatment with UPA has been proven safe since it does not compromise at any moment patient fertility, and no teratogenic effects have been described after therapy cessation [6–8].

GnRH agonists were the only treatment indicated for uterine myomatosis until recently, but patients could experience hot flushes and bone decalcification after treatments longer than 2-3 months because of the estrogen levels suppression [5].

The levonorgestrel intrauterine device has also been used to effectively control bleeding, but it does not have a short-term effect on myoma size [3]. Additionally, its use is out of label and in patients with submucosal myomas is not recommended, having shown a high rate of expulsion.

The use of UPA is a promising option in the treatment of myomas, as it has been found to control metrorrhagia and decrease the size of myomas within weeks, without relevant side effects. UPA has also been shown to be safe from a point of view of reproductive health.

## 4. Conclusion

The clinical case presented here highlights the beneficial effects of UPA in the control and preoperative treatment of myomas. The improvement of the anemia reflected an adequate control of the menometrorrhagia, with amenorrhea reported by the patient starting in the first few days after UPA administration.

The remarkable reduction in size of the myomas allowed surgery optimization, as it reduced the area of uterine wall involved. An optimal cleavage angle for enucleation and little blood loss during myomectomy was found as well.

The time that lapsed in efforts to achieve pregnancy decreased remarkably, only of 6 months after myomectomy. Pregnancy was standard in length and resulted in normal fetal weight and health.

In summary, we report a highly positive impact of UPA treatment prior to myomectomy in women of childbearing age, as it improves the clinical symptomatology of myomas in the preoperative period, facilitates and optimizes the surgery, and reaches the objective of better reproductive prospects for the patient.
